# Immunomodulatory and biological properties of helminth-derived small molecules: Potential applications in diagnostics and therapeutics

**DOI:** 10.3389/fpara.2022.984152

**Published:** 2022-09-09

**Authors:** Karma Yeshi, Roland Ruscher, Alex Loukas, Phurpa Wangchuk

**Affiliations:** Centre for Molecular Therapeutics, Australian Institute of Tropical Health and Medicine (AITHM), James Cook University, Cairns, QLD, Australia

**Keywords:** helminths, immunomodulatory, small molecules, excretory-secretory products, biological activities, pharmacological properties, metabolomics

## Abstract

Parasitic helminths secrete and excrete a vast array of molecules known to help skew or suppress the host’s immune response, thereby establishing a niche for sustained parasite maintenance. Indeed, the immunomodulatory potency of helminths is attributed mainly to excretory/secretory products (ESPs). The ESPs of helminths and the identified small molecules (SM) are reported to have diverse biological and pharmacological properties. The available literature reports only limited metabolites, and the identity of many metabolites remains unknown due to limitations in the identification protocols and helminth-specific compound libraries. Many metabolites are known to be involved in host-parasite interactions and pathogenicity. For example, fatty acids (e.g., stearic acid) detected in the infective stages of helminths are known to have a role in host interaction through facilitating successful penetration and migration inside the host. Moreover, excreted/secreted SM detected in helminth species are found to possess various biological properties, including anti-inflammatory activities, suggesting their potential in developing immunomodulatory drugs. For example, helminths-derived somatic tissue extracts and whole crude ESPs showed anti-inflammatory properties by inhibiting the secretion of proinflammatory cytokines from human peripheral blood mononuclear cells and suppressing the pathology in chemically-induced experimental mice model of colitis. Unlike bigger molecules like proteins, SM are ideal candidates for drug development since they are small structures, malleable, and lack immunogenicity. Future studies should strive toward identifying unknown SM and isolating the under-explored niche of helminth metabolites using the latest metabolomics technologies and associated software, which hold potential keys for finding new diagnostics and novel therapeutics.

## Introduction

Helminths, the cause of neglected helminthiases, are comprised of two major phyla, namely nematodes (roundworms) and platyhelminths (flatworms) (Hotez et al., 2008). Nematodes include intestinal soil-transmitted helminths (STHs) such as roundworms (e.g., *Ascaris lumbricoides*), whipworms (e.g., *Trichuris trichiura*), hookworms (e.g., *Ancylostoma* spp., and *Necator americanus*), and tissue or intestinal nematode (e.g., *Trichinella* spp.); and the filarial worms that cause lymphatic filariasis and onchocerciasis. Platyhelminths include trematodes such as blood-flukes (schistosomes) and cestodes such as tapeworms that cause cysticercosis. These parasites have been associated with humans since they were hominids, with an intimate evolutionary relationship that harks back at least two million years, and they are thus considered “heirloom parasites” (Araujo et al., 2013).

Intestinal nematodes have long been recognized as a public health burden contributing to anaemia, digestive diseases, and stunted growth (Sanchez et al., 2014). The exact global statistics for STH infections and associated morbidity are still elusive due to the inadequacy of up-to-date epidemiological data and the non-specificity of clinical signs of STH infections (De Silva et al., 2003; Brooker, 2010). According to the World Health Organisation (WHO), more than 1.5 billion people (approximately 24% of the world’s population) are infected with STHs alone, mainly affecting sub-Saharan Africa, the Americas, China, and East Asia, with children being the primary victims (WHO, 2020). More than 267 million preschool-age children and 568 million school-age children live in parasite-endemic areas needing treatment and preventive interventions.

As per the Global Burden of Disease study conducted in 2010, chronic parasitic worm infections significantly contribute to disability-adjusted life years (DALYs) lost, causing health and economic losses to billions of people worldwide (Murray et al., 2012; Vos et al., 2012). As of 2010, STHs have caused 5.2 million DALYs, schistosomiasis (3.3 million), lymphatic filariasis (2.8 million), and onchocerciasis (0.5 million) (Murray et al., 2012). However, in 2017, the estimated global burden of STHs dropped to 1.9 million DALYS (Kyu et al., 2018). According to Montresor et al. (2020), more than one billion people worldwide are still affected by STHs (Montresor et al., 2020). Currently, only a few WHO-recommended anthelmintic drugs, such as albendazole, mebendazole, and ivermectin, are used to treat STH infections (WHO, 2020). Despite repeated deworming and mass-drug administration programs in many countries, individuals in endemic areas are susceptible to re-infection. No vaccine is available for any human helminthiasis, and its development has been confounded by the complex life cycles of parasitic worms. Moreover, we do not fully understand the cues that these parasites require from the host to trigger developmental changes during their complicated lifecycles.

It is indisputable that chronic parasitic helminth infections are a major human health burden, but at the same time, epidemiological evidence supports the “Old Friends” hypothesis, notably through the observation that the complete elimination of helminths from defined human populations is associated with an increased incidence of inflammatory diseases and immune dysfunction (Rook, 2012; Rook, 2013). Although this link has not yet established cause and effect, there is clear evidence that helminth infections suppress the development of some inflammatory conditions, which is discussed in later sections. The “Old Friends” hypothesis (Rook, 2007) describes the requirement for early exposure to organisms like STHs, microbes, and environmental factors to prime essential immune development during childhood and ensure the optimal balance between effector and regulatory arms of the immune system. These immunomodulatory strategies mean that individual parasites can colonise a host for many years while preventing the development of sterilising protective immunity and suppressing the onset of inappropriate immune responses to innocuous antigens (McSorley et al., 2011; McSorley et al., 2013). Indeed, hookworms are one of the most adept infectious agents at driving regulatory responses (Chazot et al., 1994). It is well-known that the incidence of inflammatory diseases, including inflammatory bowel disease (IBD), is highest in countries where endemic exposure to helminths is lowest (Weinstock and Elliott, 2009; Rook, 2012; Molodecky et al., 2012). Extensive studies show that as a population urbanises and exposure to helminths is reduced, an increase in the incidence of inflammatory disease is observed (Prideaux et al., 2012; Ahuja and Tandon, 2010). The implication of helminths in this process is documented in mass drug administration programs, where an increase in immune dysfunction disorders soon follows the removal of helminths (Flohr et al., 2006). In a study by Kabeerdoss *et al.* (2011) (Kabeerdoss et al., 2011) of Crohn’s disease patients in India, individuals depleted of hookworms were more likely to have Crohn’s disease than matched infected subjects. In South Africa, childhood exposure to helminths was shown to be protective against IBD (Chu et al., 2013), and in Papua New Guinea, exposure to intestinal parasites protected infected subjects against the development of allergic rhinitis (Blount et al., 2009). There are also small open-label studies showing disease improvement in adult IBD patients (e.g., Crohn’s disease) when treated with tolerable numbers of live helminths (e.g., human hookworm *N. americanus* infective larvae) (Croese et al., 2006; Helmby, 2015). Inspired by these beneficial health effects, many patients suffering from autoimmune and metabolic diseases, especially IBD, have resorted to an unregulated and risky self-treatment modality called helminth therapy propagated by the availability of helminths online (Zhang and Gems, 2021).

The key to the helminth’s ability to successfully evade the host immune system’s anti-helminth responses and establish a chronic infection while rendering protection to their host against various inflammatory conditions lies in their excretory/secretory products (ESPs) (Loukas et al., 2016; Eichenberger et al., 2018; Wangchuk et al., 2019). Helminths actively induce tolerance (both local and systemic) by producing ESPs that interact with the host immune system. The ESPs comprise proteins, peptides, organic acids, lipids, carbohydrates, and nucleic acids (Maizels et al., 2018). Some of these molecules are homologous to host molecules and can skew the host’s immune response by mimicking immunologically relevant proteins or miRNAs that target the host’s gene expression (Buck et al., 2014; Smyth et al., 2018). Other helminth-derived molecules are unique to the parasites and help them initiate tissue damage and establish infection (Periago and Bethony, 2012; Flynn et al., 2019). Such helminth-specific biomolecules have also been explored widely as biomarkers for developing diagnostics and targets for developing vaccines and drugs (Hamed et al., 2011; Ryan et al., 2020; Whitman et al., 2021). Numerous reviews (McSorley et al., 2013; Maizels and McSorley, 2016; Maizels et al., 2018; Zakeri et al., 2018; Ryan et al., 2020) have substantially discussed the nature and functions of many helminth-derived macromolecules, including secreted proteins, peptides, and extracellular vesicles. Recently, work on helminth-derived small molecules (SM) has gained traction (Bobardt et al., 2020). However, to our knowledge, there is no comprehensive review article on the SM components of helminths.

Small molecules are chemical compounds whose size/molecular weights are less than one kilodalton (kDa) (Makurvet, 2021). Herein, we have collected published information on SM (identified from helminths) and their immunomodulatory potential from PubMed, Scopus, MEDLINE Ovid, Google Scholar Databases, and journal articles and analysed their contents. To retrieve relevant published information, we used the keywords “small molecules,” “helminths small molecules,” “immunomodulatory,” “helminth-derived small molecules,” “host-parasite interaction,” “helminth metabolites,” “lipidomics,” “helminth therapy,” “metabolomics,” “targeted metabolomics,” “untargeted metabolomics,” “inflammation,” “inflammatory bowel diseases,” “autoimmune disease,” “inflammatory cytokines,” “anti-inflammatory,” “helminthiases,” “clinical trials,” “host-parasite interaction,” “biomarkers,” “down-regulation,” “up-regulation,” “excretory/secretory products,” and names of different helminth species.” We have conducted a narrative review of the available literature and presented SM identified from helminths and their reported biological activities, exploring their potential scope in diagnostics and therapeutics development.

## Host-parasite interaction and immunomodulation

**Figure 1:**
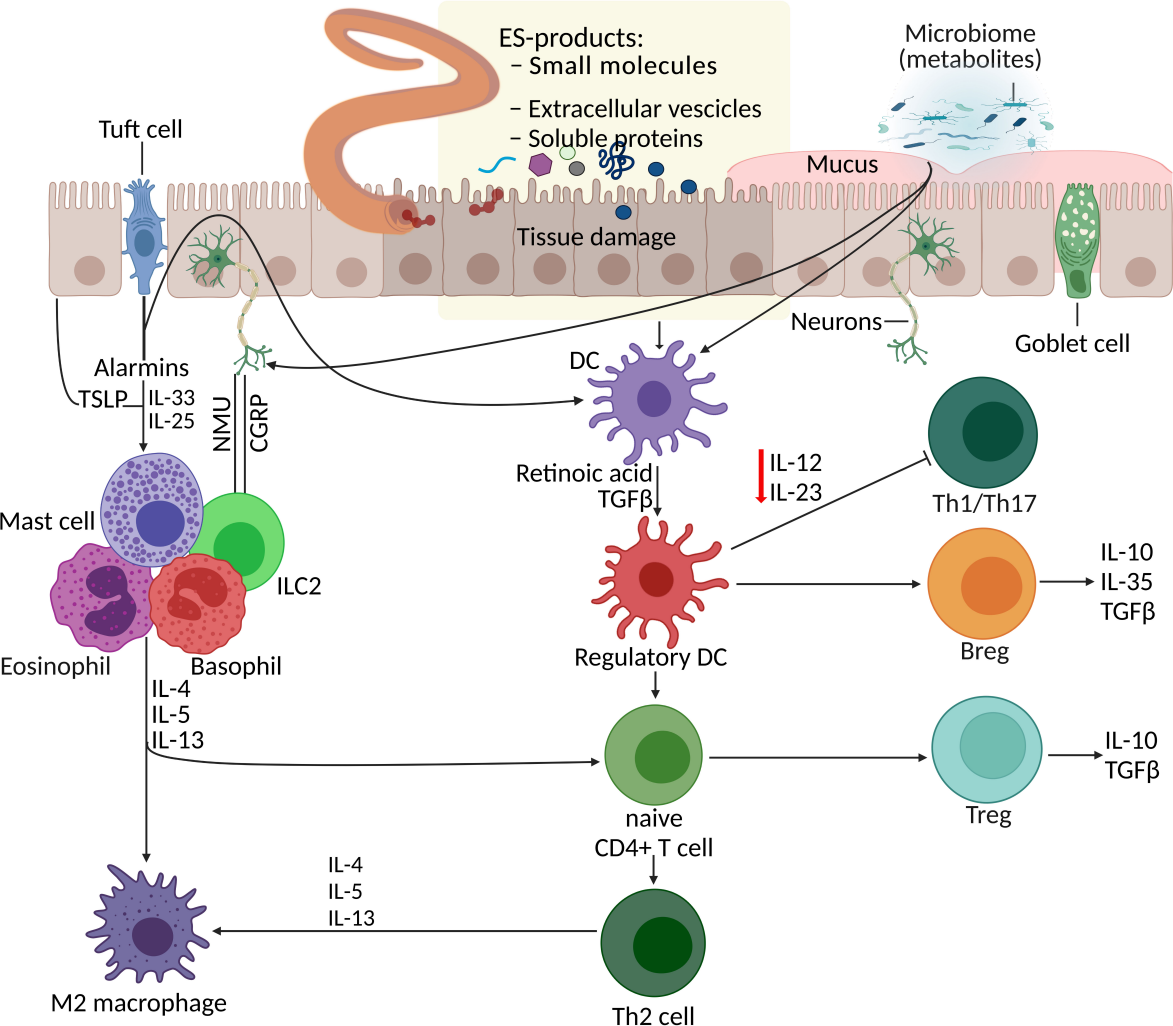
Parasite–host interaction: manipulation of the host immune system by helminth excretory/secretory products (ESPs). Helminth infection together with additional events and signals (such as worm ESPs, microbiota metabolites, and alarmins from tissue damage) initiate processes including the differentiation of Th2 cells, Treg expansion and responses, macrophage polarisation, and mucus production. DCs respond to various stimuli (alarmins, ESPs, and metabolites) and promote development of Treg, Breg, and Th2 cells, and inhibit Th1 and Th17 cell responses. Additionally, alarmins (TSLP, IL-33, IL-25) released by tuft cells and other epithelial cells following tissue damage, act on granulocytes (mast cells, basophils, and eosinophils) and ILC2s, consequently enhancing the production of type 2 cytokines. Gut sensory neurons also influence this immune network upon receiving signals from helminths and microbes by releasing neuropeptides (NMU- neuropeptide neuromedin U and CGRP-calcitonin gene-related peptide) which directly effects the ILC2 responses. APR, aspartic protease; Breg, regulatory B cell; CGRP, calcitonin gene-related protein; DC, dendritic cell; ESP, excretory/secretory product; GST, glutathione-S-transferase; ILC2, type 2 innate lymphoid cell; NMU, neuromedin U; serpin, serine protease inhibitor; Th2, T helper type 2, TLSP, thymic stromal lymphopoietin; Treg, regulatory T cell.

Parasitic helminths are believed to have coevolved with humans over millennia, continuously refining and improving their mechanisms to skew or suppress the host’s immune response to establish a sustained niche inside their host. They do so by secreting or excreting a vast array of molecules, including metabolites. Dendritic cells (DCs) respond to ESPs and also microbiome metabolites, consequently promoting the development of regulatory T cells (Treg), regulatory B cells (Breg), and T helper type 2 (Th2) cells and inhibiting Th1 and Th17 cell responses (**Figure 1**). For instance, *Trichinella spiralis* adult and muscle larva-derived ESPs activated bone marrow-derived dendritic cells enabled naïve mice to acquire Treg cells differentiation (Sun et al., 2019), suggesting that *T. spiralis* ESPs might stimulate differentiation of the host’s Treg cells *via* dendritic cells activation favoring parasite survival inside the host. Helminths residing in the human gut induce tolerogenic DCs, alternatively activated macrophages, and Treg cells, which produce suppressor cytokines that help keep inflammatory T cells and their effector molecules in check (Loukas et al., 2016). Helminths-induced type 2 immune response causes profound changes in the host tissue physiology, hyperplasia of goblet cells (Miller and Nawa, 1979), and hypercontractility of smooth muscles (Castro et al., 1976). Additionally, the innate lymphoid cell (ILC) family and T and B lymphocytes are involved in host defence, barrier integrity, and tissue homeostasis and repair (Artis and Spits, 2015). Alarmins such as thymic stromal lymphopoietin (TSLP), IL-33, and IL-25 released by tuft cells (**Figure 1**) and other epithelial cells following tissue damage act on granulocytes (mast cells, basophils, and eosinophils) and tissue-resident group 2 ILCs (ILCs-2), consequently enhancing the production of type 2 cytokines, including IL-13 (Grencis, 2015; von Moltke et al., 2016).

As helminths (both nematodes and platyhelminths) transition from their free-living stage to parasitism, they require long-lasting niches, including intermediate hosts, to complete their life cycle (Hotez et al., 2008). In the course of their journey, they either secrete or excrete many molecules that help them enter their host, invade tissues, shield them from the host’s immune assault and thus, establish chronic infections (reviewed elsewhere (Maizels et al., 1993; Maizels and Yazdanbakhsh, 2003; Dzik, 2006; Maizels and McSorley, 2016; Ryan et al., 2020)). For instance, skin is the first immune barrier *Schistosoma mansoni* has to pass through. Proteolytic factors, including various serine proteases, are reported to be responsible for skin penetration (Marikovsky et al., 1988; Chavez-Olortegu et al., 1992). Among many serine proteases, the 28/30kDa variant called SmCE (*S. mansoni* cercarial Elastase) is considered most crucial as inhibition (by inhibitor succinyl-alanyl-alanyl-prolyl-phenylalanine chloromethyl ketone, AAPF-CMK) of this protease reduces skin penetration up to 80% (Salter et al., 2000). After successful skin penetration, one of the mechanisms that favor the survival of lung stage schistosomula is using their surface-associated peroxiredoxin 1 to overcome oxidative stress scavenging surrounding hydrogen peroxide (Kumagai et al., 2009). Surface-bound enolases of schistosomula in both *S. japonicum* and *S. mansoni* are associated with a fibrinolytic activity which prevents clot formation in their vicinity (Figueiredo et al., 2015). Glycoproteins such as alpha-1 (also known as IL-4 inducing principle of *S. mansoni* eggs, IPSE) and omega-1 are known to involve in skewing T cell response in granulomas (Th2 type response) while migrating through the intestine towards the lumen (Everts et al., 2009; Haeberlein et al., 2017). The interaction mechanism with host tissues is complex and hard to dissect at the molecular level, partly due to the lack of suitable animal models (Loukas and Prociv, 2001), and the roles of many bigger molecules, particularly proteins, are yet to be understood. This review highlights known or potential roles of SM either secreted and/or excreted by various helminths in their life cycle stages, including while interacting with their respective hosts (**Table 1**). Octadecanoic acid secreted by the infective third-stage larvae (L3) of *Nippostrongylus brasiliensis* (a model organism for human hookworms) is known to help to lyse the host’s red blood cells, and similarly, in *Haemonchus contortus* (Ward, 1982; Yeshi et al., 2020). Another small molecule, succinic acid, identified from the ESP of adult *N. brasiliensis*, can induce intestinal tuft cells to initiate Th2 responses (Wangchuk et al., 2019). Succinic acid is thought to be energy source for helminths during adaptations toward anaerobiosis (Saz, 1981). Succinic acid is also detected as a dominant metabolite from the ESPs of adult-stage *Trichuris muris* (a model organism for the human-dwelling *T. trichiura*) (Wangchuk et al., 2019).

**Table:** TABLE 1 Reported biological/immunomodulatory properties of MSI level 1 confirmed small molecules from helminths.

Small molecules	Chemical classes^*^	Helminth species in which SM were detected	Reported biological/immunomodulatory properties
Acetate	Carboxylic acid and derivatives	*A. caninum*, *N. brasiliensis*, *T. muris*, *T. canis*, *A. lumbricoides*	Anti-inflammatory (Tedelind et al., 2007)
Adenine	Imidazopyrimidines	*N. brasiliensis, N. americanus, T. muris*	Anti-inflammatory (Fukuda et al., 2017)
Adenosine	Purine nucleosides	*N. brasiliensis, N. americanus, T. muris*	Anti-inflammatory (Yang et al., 2006)
Adenosine 5’-monophosphate	Purine nucleotides	*N. brasiliensis, N. americanus, T. muris*	Anti-inflammatory (Sag et al., 2008; Yamaguchi et al., 2014)
Arachidonic acid	Fatty acyls	*N. brasiliensis, T. muris, D. caninum, T. canis*	It regulates blood clotting, including platelet aggregation; helps in smooth muscle contraction, leukocyte chemotaxis, inflammatory cytokine production and immune function (Wishart et al., 2018)
Azelaic acid	Fatty acyls	*N. brasiliensis, T. muris*	Anti-inflammatory (Mastrofrancesco et al., 2010)
Betaine	Carboxylic acid and derivatives	*N. brasiliensis, N. americanus, T. muris*	Neuroprotective (Singhal et al., 2020); improves intestinal barrier function (Wu et al., 2020); hepatoprotective (Khodayar et al., 2020); anti-inflammatory (Detopoulou et al., 2008)
Butyrate	Faty acyls	*A. caninum*, *N. brasiliensis*, *T. muris*, *T. canis*, *A. lumbricoides*	Anti-inflammatory (Andoh et al., 1999; Kovarik et al., 2011)
Choline	Organonitrogen compounds	*N. brasiliensis, N. americanus, T. muris*	Anti-inflammatory (Detopoulou et al., 2008)
Citric acid	Carboxylic acid and derivatives	*N. brasiliensis, T. muris, A. caninum, D. caninum, T. canis*	Cardioprotective, antimicrobial (Tang et al., 2013).
Deoxyadenosine	Purine nucleosides	*N. brasiliensis, T. muris*	Cell growth inhibitor and cytotoxic (Ullman et al., 1978)
Docosahexaenoic acid	Faty acyls	*N. brasiliensis, T. muris, D. caninum, T. canis*	Anti-inflammatory (Kuda, 2017); cardioprotective (Wishart et al., 2009)
Fructose	Organooxygen compounds	*T. canis, T. muris, D. caninum.*	Associated with hypertension and hepatic disorders (Wishart et al., 2018); pro-inflammatory (Glushakova et al., 2008).
Glucosamine	Organooxygen compounds	*N. brasiliensis, T. muris, T. canis*	Used in osteoarthritis treatment (Largo et al., 2003); anti-inflammatory (Wishart et al., 2018).
Glycerol	Organooxygen compounds	*N. brasiliensis, T. muris, A. caninum, D. caninum, T. canis*	Anti-inflammatory (Szel et al., 2015)
γ-aminobutyric acid	Carboxylic acid and derivatives	*A. caninum, D. caninum, T. canis*	Antidiabetic, regulate blood pressure (Wishart et al., 2009); anti-inflammatory (wound-healing agent) (Han et al., 2007)
Homogentisate	Benzene and substituted derivatives	*N. brasiliensis, T. muris*	Pro-inflammatory (Hegedus and Nayak, 1994; Spreafico et al., 2013)
Hypoxanthine	Imidazopyrimidines	*N. brasiliensis, N. americanus, T. muris*	Anti-inflammatory (Lee et al., 2018)
Inosine	Purine nucleosides	*N. brasiliensis, N. americanus, T. muris*	Anti-inflammatory (Hasko et al., 2000; Liaudet et al., 2001)
Lactic acid	Hydroxy acids and derivatives	*N. brasiliensis, T. canis, A. caninum, D. caninum.*	Anti-inflammatory and immunosuppressant (Hearps et al., 2017)
Lauric acid (dodecanoic acid)	Fatty acyls	*N. brasiliensis, T. muris, A. caninum, D. caninum, T. canis*	Anti-inflammatory, antibacterial (Huang et al., 2014); Facilitate interaction with their hosts (Ferreira et al., 2014)
Linoleic acid	Fatty acyls	*N. brasiliensis, T. muris, A. caninum, D. caninum, T. canis*	Anti-inflammatory (Rodrigues et al., 2012); Helps parasites to migrate successfully inside their host (Giera et al., 2018)
L-Arginine	Carboxylic acid and derivatives	*N. brasiliensis, N. americanus, T. muris*	Anti-inflammatory (Hnia et al., 2008; Coburn et al., 2012; Wu et al., 2016)
L-Alanine	Carboxylic acid and derivatives	*N. brasiliensis, N. americanus, T. muris, A. caninum, D. caninum, T. canis*	Anti-inflammatory (Naügeli et al., 2014; Raizel et al., 2016; Coqueiro et al., 2017); dissolves kidney stones in experimental animals (Wishart et al., 2009)
L-Aspartate	Carboxylic acid and derivatives	*N. brasiliensis, N. americanus, T. muris, A. caninum, D. caninum.*	Anti-inflammatory and neuroprotective (Boccella et al., 2015; Afraei et al., 2017); excitatory neurotransmitter (Wishart et al., 2009)
L-Carnitine	Organonitrogen compounds	*N. brasiliensis, N. americanus, T. muris*	Anti-inflammatory (Lee et al., 2015) and antioxidant (Keskin et al., 2015)
L-Citrulline	Carboxylic acid and derivatives	*N. brasiliensis, T. muris, N. americanus*	Anti-inflammatory and antioxidant (Ham et al., 2015; Lee et al., 2018; Darabi et al., 2019)
L-Glutamate	Carboxylic acid and derivatives	*N. brasiliensis, N. americanus, T. muris*	Antioxidant (Jiao et al., 2015)
L-Glutamine	Carboxylic acid and derivatives	*N. brasiliensis, N. americanus, T. muris*	Anti-inflammatory (Naügeli et al., 2014; Raizel et al., 2016; Coqueiro et al., 2017); maintains gut barrier function and reduces the symptoms of IBS (irritable bowel syndrome) (Wishart et al., 2009)
L-Histidine	Carboxylic acid and derivatives	*N. brasiliensis, N. americanus, T. muris, A. caninum*	Anti-inflammatory (Hasegawa et al., 2012)
L-Leucine	Carboxylic acid and derivatives	*N. brasiliensis, N. americanus, T. muris*	Analgesic and anti-inflammatory (Saxena et al., 1984; Kato et al., 2016)
L-Lysine	Carboxylic acid and derivatives	*N. brasiliensis, N. americanus, T. muris, A. caninum*	Anti-inflammatory (He et al., 2018; Han et al., 2018)
L-Methionine	Carboxylic acid and derivatives	*N. brasiliensis, N. americanus, T. muris, A. caninum, D. caninum, T. canis*	Anti-inflammatory (Unnikrishnan and Rao, 1990) and antioxidant (Derakhshanfar et al., 2009)
L-Phenylalanine	Carboxylic acid and derivatives	*N. brasiliensis, N. americanus, T. muris, A. caninum, D. caninum, T. canis*	Antidiabetic (Alamshah et al., 2017)
L-Proline	Carboxylic acid and derivatives	*N. brasiliensis, T. muris, N. americanus, A. caninum*	Anti-inflammatory (Faure et al., 2006)
L-Serine	Carboxylic acid and derivatives	*N. brasiliensis, T. muris, N. americanus, A. caninum*	Modulates adaptive immunity by controlling T cell proliferative capacity (Ma et al., 2017); colon protection and mucosal healing (Faure et al., 2006)
L-Threonine	Carboxylic acid and derivatives	*N. brasiliensis, T. muris, N. americanus, A. caninum*	Anti-inflammatory (Dong et al., 2017; Gaifem et al., 2018)
L-Tryptophan	Indoles and derivatives	*N. brasiliensis, T. muris, N. americanus, A. caninum, T. canis*	Anti-inflammatory (Mine and Zhang, 2015; Yue et al., 2017; Islam et al., 2017)
L-Valine	Carboxylic acid and derivatives	*N. brasiliensis, T. muris, N. americanus, A. caninum*	Anti-inflammatory (Mine and Zhang, 2015)
Maleic acid	Carboxylic acid and derivatives	*N. brasiliensis, T. muris*	Inflammatory/Cytotoxic (Andersen, 2007)
Malic acid	Hydroxy acids and derivatives	*N. brasiliensis, T. muris, A. caninum, D. caninum, T. canis*	Anti-inflammatory and anti-aggregant (Tang et al., 2013).
Mannitol	Organooxygen compounds	*N. brasiliensis, N. americanus, T. muris, A. caninum, D. caninum, T. canis*	Anti-edema and diuretic (Cavone et al., 2012).
Methionine	Carboxylic acid and derivatives	*N. brasiliensis, N. americanus, T. muris, A. caninum, D. caninum, T. canis*	Anti-inflammatory (Unnikrishnan and Rao, 1990); antioxidant (Derakhshanfar et al., 2009); associated with intellectual disability in infants, delays motor skills, sluggishness, liver problems and muscle weakness (Wishart et al., 2009)
*myo*-inositol	Organooxygen compounds	*N. brasiliensis, T. muris, A. caninum, D. caninum, T. canis*	Useful for treating polycystic ovary syndrome (Wishart et al., 2009)
N-Acetylputrescine	Carboximidic acids and derivatives	*N. brasiliensis, N. americanus, T. muris*	Lung cancer biomarker (Liu et al., 2017)
N6,N6,N6-Trimethyl-L-lysine	Carboxylic acid and derivatives	*N. brasiliensis, T. muris, N. americanus*	Cardiovascular disease biomarker (Li et al., 2018)
Oleic acid	Fatty acyls	*N. brasiliensis, T. muris*	Anti-inflammatory (Rodrigues et al., 2012)
Palmitic acid	Fatty acyls	*N. brasiliensis, T. muris, A. caninum, D. caninum, T. canis*	Anti-inflammatory (Aparna et al., 2012)
Propionate	Carboxylic acid and derivatives	*A. caninum*, *N. brasiliensis*, *T. muris*, *T. canis*, *A. lumbricoides*	Anti-inflammatory (Tedelind et al., 2007)
Pterin	Pteridines and derivatives	*N. brasiliensis*	Biomarker of exercise-induced stress (Lindsay and Gieseg, 2020)
Scyllo-inositol	Organooxygen compounds	*D. caninum, T. canis*	Neuroprotective (Wishart et al., 2009)
Sorbitol	Organooxygen compounds	*N. brasiliensis, T. canis, A. caninum, D. caninum.*	Laxatives and irrigating solution in surgeries (Wishart et al., 2009)
Stearic acid (octadecanoic acid)	Fatty acyls	*N. brasiliensis, T. muris, A. caninum, D. caninum, T. canis*	Anti-inflammatory (Ahmad et al., 2015); anticancer and cardioprotective (Chajes et al., 1999; Kris-Etherton et al., 2005); Alters physical properties of the hosts’ red blood cells membrane and causes lysis (Ward, 1982; Yeshi et al., 2020)
Succinate	Carboxylic acid and derivatives	*N. brasiliensis, N. americanus, T. muris, A. caninum, D. caninum, T. canis*	Anti-inflammatory (Littlewood-Evans et al., 2016; Lei et al., 2018); Induces intestinal tuft cells to initiate Th2 response (Wangchuk et al., 2019)
Talose	Lactones	*N. brasiliensis, T. canis, A. caninum, D. caninum.*	Cell growth inhibitor (C. elegans) (Sakoguchi et al., 2016)
Tyrosine	Carboxylic acid and derivatives	*N. brasiliensis, N. americanus, T. muris, A. caninum, D. caninum, T. canis*	Neuroprotective (Wishart et al., 2018).
Uridine	Pyrimidine nucleosides	*T. muris, A. caninum*	Anti-inflammatory (Evaldsson et al., 2007)
Urocanate	Azoles	*N. brasiliensis, N. americanus, T. muris*	Chemoattractant (Safer et al., 2007)
Xanthine	Imidazopyrimidines	*N. brasiliensis, N. americanus, T. muris*	Proinflammatory (Joshi et al., 2018)
2-Oxoglutarate	Keto acids and derivatives	*N. brasiliensis, N. americanus, T. muris*	Anti-inflammatory (He et al., 2017) and antioxidant (Velvizhi et al., 2002)
4-Hydroxybenzoate	Benzene and substituted derivatives	*N. brasiliensis, N. americanus, T. muris*	Neuroprotective (Winter et al., 2017)
5-Aminolevulinate	Carboxylic acid and derivatives	*N. brasiliensis, N. americanus, T. muris*	Anti-inflammatory (Fujino et al., 2016)
5-Oxoproline	Carboxylic acid and derivatives	*N. brasiliensis, N. americanus, T. muris, A. caninum, D. caninum, T. canis*	Neuroprotective (Largo et al., 2003; Pederzolli et al., 2010)
2,5-Dihydroxybenzoate	Carboxylic acids	*N. brasiliensis*	Anticancer activity (Kalinowska et al., 2018)
3’,5’-Cyclic AMP	Purine nucleotides	*N. brasiliensis, N. americanus, T. muris*	Anti-inflammatory (Oldenburger et al., 2012)

MSI-level-1 confirmed small molecules (SM) are those metabolites/compounds whose structure is fully validated with reference standards based on mass spectrometry, tandem mass spectrometry, and retention time matching. *Chemical classes as described in HMDB and PubMed.

## Old friends/hygiene hypothesis: evidence from helminth therapy

From an evolutionary viewpoint, the coevolution of humans and helminths may have shaped our metabolism and organ functions (Stilling et al., 2014). Infection with our ‘old friends’ – helminths – is believed to have shaped our immune system to facilitate co-existence (Rook et al., 2014; Versini et al., 2015; Longman and Littman, 2015; Filyk and Osborne, 2016), and there is evidence that helminths can protect us from a broad spectrum of immune-mediated inflammatory diseases (IMIDs). These changes to the immune system have often benefited hosts by reducing overall inflammation. Moreover, as per the hygiene hypothesis (first proposed by Strachan) (Strachan, 1989), reduced contact with infectious agents (particularly those that co-evolved with us) due to improved sanitation, lifestyle changes (including dietary habits) are attributed to the alarming rise in IMIDs, including metabolic syndrome (e.g., obesity, insulin resistance, hypertension, and atherogenic dyslipidemia) (Grundy et al., 2005; Huang, 2009; Rook et al., 2014). Several studies have reported that helminths promote the host’s gut health/influence microbiome composition (evident from their fecal microbiome) (Lee et al., 2014; Jenkins et al., 2017; Jenkins et al., 2018; Rosa et al., 2018; Schneeberger et al., 2018; Gordon et al., 2020; Mejia et al., 2020), including increased alpha diversity (multispecies-helminth infected individuals with greater diversity than single species-infected individuals). The influence on microbiome composition is found to be specific to helminth species and the host’s anatomical niche (e.g., observed strong influence by helminths in the large intestine such as *Enterobius vermicularis* and *Trichuris trichiura*). Studies have also shown that infection with various helminth species can reduce the severity of diseases, including chemically-induced colitis (Fox et al., 2000; Khan et al., 2002; Elliott et al., 2004; Moreels et al., 2004; Hunter et al., 2005) and high fat diet-induced obesity (Su et al., 2018; Su et al., 2020), and such protection is partially attributed to Th2-dependent, M2 macrophage-mediated change in the gut microbiome (Durack and Lynch, 2019; Su et al., 2020). For instance, targeted removal of the bacterial family Enterobacteriaceae reduced inflammation in mice (Zhu et al., 2018). Reviews done by Brussow (2019) (Brussow, 2020), Kupritz et al. (2021) (Kupritz et al., 2021), and Durack and Lynch (2019) have more detailed updates on the helminth-induced human gut microbiome composition and their benefits and therapeutic potential. The human gut microbiome is also said to be influenced by deworming (the clearance effect) and anthelmintic drugs (the treatment effect) (Kupritz et al., 2021). For instance, a study conducted in a rural Kenyan village observed that the clearance of STHs (e.g., *A. lumbricoides* and *N. americanus*) using albendazole therapy led to a significant increase in Clostridials and reduction in Enterobacteriales in the gut microbiome of participants when compared with their age-matched controls (Easton et al., 2019). A similar finding was also reported from the cross-sectional studies between Indonesia and Liberia, where both countries showed a significant association of 12 bacterial taxa with STH infections, including *Olsenella* (associated with reduced gut inflammation), which decreases in numbers after deworming (Rosa et al., 2018). It is difficult to discriminate the influence between the clearance and treatment effect, and further, the change in microbiome composition is mostly significant only in treated subjects who remain infected after the treatment (Martin et al., 2018; Martin et al., 2019). Perturbations in the gut microbiome (e.g., the imbalance between harmful and protective bacteria) or dysbiosis characterised by, for instance, reduced diversity of short-chain fatty acids (SCFAs) producers and enriched proinflammatory microbes (e.g., H_2_S producers) is said to be responsible for the pathogenesis of IBD, and it is reviewed elsewhere (Kaur et al., 2011; Sultan et al., 2021). For example, the lack of certain symbiotic species (e.g., *Faecalibacterium prausnitzii*) is said to be responsible for the recurrence of Crohn’s disease as mice supplemented with this organism experienced reduced inflammation after inducing colitis chemically (Sokol et al., 2008). A reduced amount of *F. prausnitzii* is also linked to relapse of Ulcerative colitis (Werkman et al., 2018). However, the causal effect of dysbiosis on IBD and other diseases is still inconclusive, as dysbiosis is even observed in the fecal samples from healthy relatives of IBD patients (Van de Merwe et al., 1988). There is no cure for IMIDs, and some patients, in desperation and inspired by the above theories, have turned to alternative treatments such as helminth therapy by deliberately self-infecting with live helminths. Only two helminth species, pig whipworm *Trichuris suis* ova (TSO) and the human hookworm *N. americanus* are used for human helminth therapy.

In recent years, helminth therapy has been assessed in early phase clinical trials using TSO and *N. americanus* infective larvae against inflammatory, autoimmune, and allergic diseases (Summers, 2003; Croese et al., 2006; Blount et al., 2009), yielding both positive and negative outcomes. IBD patients (both Crohn’s disease and ulcerative colitis), when given TSO (single dose) in open-label phase I trials, not only tolerated the treatment but also achieved remission, and benefits were maintained through repeated doses (Summers, 2003; Summers et al., 2005). However, in a double-blind, placebo-controlled phase II trial of TSO in adults with active Crohn’s disease (n = 252), ingestion of 250-7500 TSO fortnightly over 12 weeks was observed to be safe without any adverse effects, but the treatment did not have an advantage over the placebo in inducing clinical remission and other immunological responses (Scholmerich et al., 2017). Moreover, the study failed to reach its clinical endpoint for both active Crohn’s disease (Scholmerich et al., 2017) and multiple sclerosis (Voldsgaard et al., 2015; Fleming et al., 2019). A meta-analysis on TSO therapy trials (Huang et al., 2018) found that the treatment did not show statistically significant benefits for IBD and suggested the need for further studies with larger sample sizes. One of the challenges of using TSO is that frequent dosing is required for human trials as *T. suis* is not adapted to develop in humans and is rapidly expelled. On the contrary, *N. americanus* is a human hookworm, and it can survive within the human body for years. Crohn’s disease patients (a few already in remission before the trial started) tolerated a low dose of *N. americanus* larvae during a phase I open label trial, where larvae were administered percutaneously (Croese et al., 2006). Even at a lower dose (initial 10 larvae, followed by a booster dose of 5 larvae at week 12), *N. americanus* larvae was partially effective over placebo when tested in 20 HLA-DQ2+ celiac disease patients (autoimmune response to dietary gluten) followed by incremental wheat challenge after 20 weeks. Patients not only tolerated the treatment, but their duodenal biopsies showed reduced inflammatory cytokines such as IFN-γ and IL-17 (McSorley et al., 2011); however, gluten challenge did not yield any difference in the clinical parameters between the treated and placebo groups (Daveson et al., 2011). In a recent larger randomised controlled trial with the increasing exposure of celiac disease patients to gluten (Croese et al., 2020), *N. americanus* larvae could not restore tolerance to sustained moderate gluten consumption (2 g/day), but hookworm-infected patients did experience reduced symptoms when gluten was given intermittently at a lower dose. In summary, there are numerous clinical trials involving live helminths, but they remain inconclusive (Elliott and Weinstock, 2017; Ryan et al., 2020). Such drawbacks could be due to difficulty controlling the quality and innocuity of the infectious agent, low doses of worms that are insufficient to achieve required immunomodulation, logistical issues with scale-up, patient compliance to the treatments, and inability to define good manufacturing standards. Moreover, persistent infection can cause various health problems, including anaemia, malnutrition, and cognitive retardation (Albonico et al., 2008), and can sometimes be fatal. For instance, Kradin et al. (2006) found sexually mature male trichurid in the cecum of pediatric Crohn’s disease patients treated with *T. suis* ova (Kradin et al., 2006). Furthermore, the pathogenicity of *Campylobacter jejuni* may increase in patients receiving *T. suis* ova treatment (Shin et al., 2004). These complications and challenges necessitate the investigation of safer molecules to develop alternative therapeutics.

In pursuit of a more palatable treatment approach, recent focus has been on ESPs of helminths, constantly secreted by adult worms into the tissues of infected hosts in their various final niches (Loukas et al., 2016; Eichenberger et al., 2018; Wangchuk et al., 2019). Studies have identified several recombinant ESP proteins that can protect mice against inflammation and weight loss in two different mouse models of colitis – the T cell-dependent TNBS (2,4,6-Trinitrobenzene sulfonic acid) model (Ruyssers et al., 2008; Buitrago et al., 2021) and the T cell-independent DSS (dextran sodium sulfate) model (Ferreira et al., 2013; Ryan et al., 2020; Wang et al., 2020). *Trichinella spiralis* 53 kDa glycoprotein (Tsp53) is anti-inflammatory (induces production of IL-4 and IL-10 in T cells) (Cvetkovic et al., 2016), and its recombinant form rTsp53 confer protection in TNBS-induced colitis in mice (Du et al., 2011). Moreover, rTsp53 can downregulate proinflammatory cytokines and increase IL-10 and TGF-β1 levels *via* inducing macrophage polarisation (M1 to M2) (Chen et al., 2016). Other *T. spiralis*-derived molecules, including cystatin-like protein (Ts-CLP) (Liu et al., 2014; Tang et al., 2015), calreticulin (Ts-CRT) (Zhao et al., 2017), cystatin (TsCstN) (Kobpornchai et al., 2020), glutathione-S-transferase (TsGST) (Jin et al., 2019), and paramyosin (Ts-Pmy) (Sun et al., 2015; Guo et al., 2016) are known to possess anti-inflammatory and immunomodulatory properties. The main immunomodulatory mechanism shown by these proteins is upregulating Treg cells to produce more IL-10 and TGF-β, thus inhibiting Th1 and Th17-mediated inflammation.

## Reported biological/immunomodulatory properties of helminth-derived small molecules

Due to their pathogenic and infectious nature, parasitic helminths can produce a wide array of immunomodulatory molecules, including proteins, glycans, nucleic acids, lipids, and SM (Maizels et al., 2018; Kahl et al., 2018). Wangchuk et al. (Wangchuk et al., 2019) reported that crude ESPs from *A. caninum* could suppress colitis pathology in a mouse model and further inhibit the secretion of inflammatory cytokines in primary human leukocytes. Ferreira et al. (Ferreira et al., 2013) also reported a similar reduced inflammatory response after administration of *A. caninum* crude ESPs in a murine colitis model by inducing a type 2 cytokine response. Therefore, we have reviewed bioactivities associated with metabolites (both polar and non-polar) identified/isolated from helminths, including literature describing metabolites’ role in the development of diagnostics/biomarkers and therapeutics. Polar metabolites, including carbohydrates, amino acids, nucleic acids, and organic acids, are hydrophilic. While non-polar metabolites, mainly lipids, are hydrophobic.

**Figure 2:**
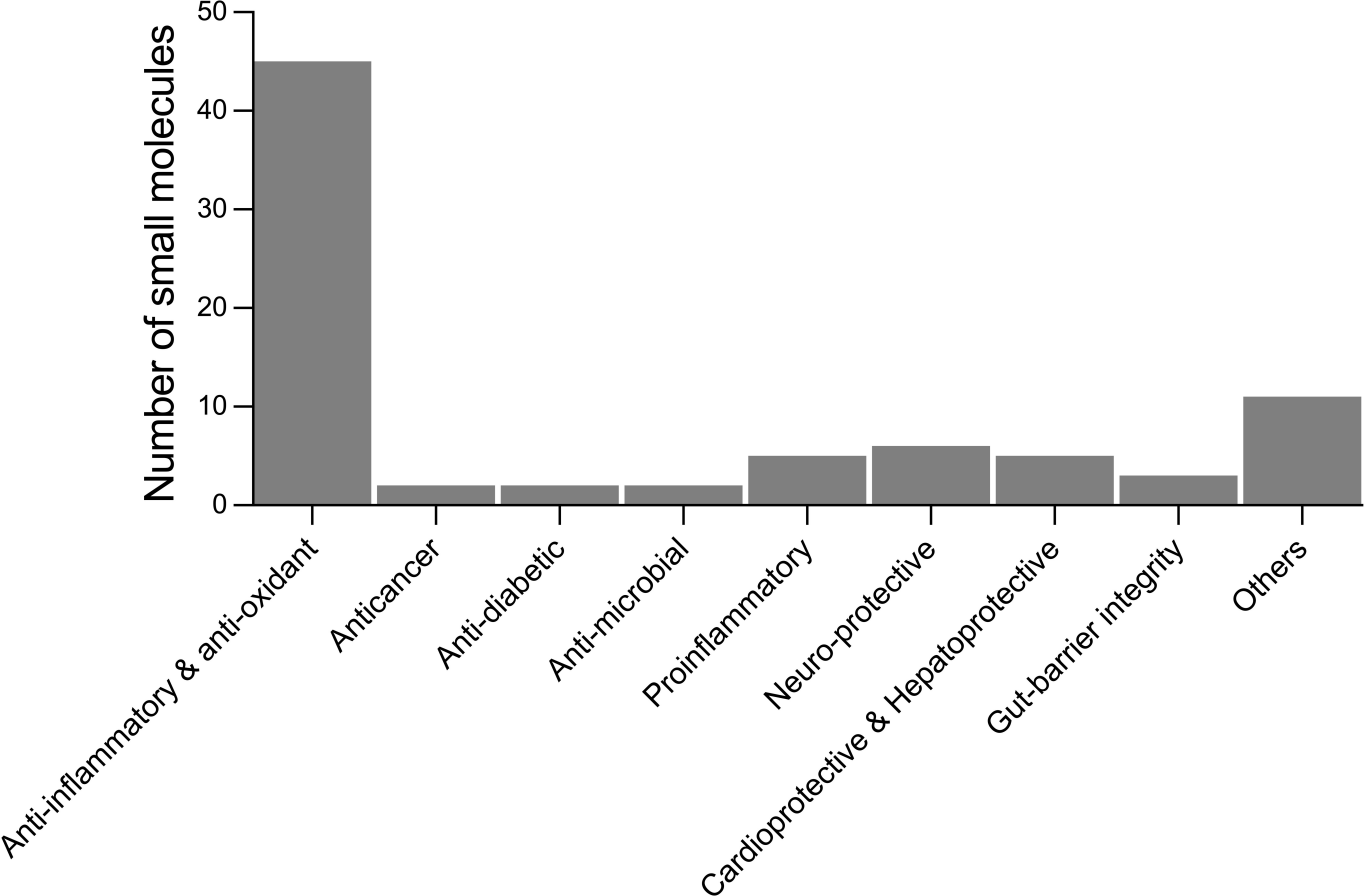
Number of small molecules identified in helminths with reported biological activities from the literature. Others include analgesics, laxatives, anti-aggregant, immuno-modulatory, cell-growth inhibitors, regulate blood clotting, treatment of osteoarthritis, polycystic ovary syndrome, and disease biomarkers.

From the literature review of helminth-derived SM reported so far, about 65 were associated with various biological activities such as anti-inflammatory and antioxidant, anti-microbial, anticancer, anti-diabetic, cardio-, hepato-, and neuro-protective, and maintaining gut-barrier integrity (**Table 1**). These 65 helminth-derived compounds were identified using metabolomics standard initiative level I identification protocols, the highest identification level achieved using standards. Most of these compounds (45 metabolites) are associated with anti-inflammatory and antioxidant properties, suggesting roles in active immune regulation (**Figure 2**). For example, major metabolites detected in the infective stages of *T. muris* and *N. brasiliensis*, such as adenine, adenosine, betaine, and choline, possess anti-inflammatory properties (see **Table 1**). Betaine and other amino acids such as glutamine and serine are known to help maintain colon homeostasis (Faure et al., 2006; Wishart et al., 2009; Wu et al., 2020). The amino acid L-serine produced by *N. americanus*, *N. brasiliensis*, *A. caninum*, and *T. muris* has been shown to modulate adaptive immunity by controlling T cell proliferative capacity (Ma et al., 2017) and is involved in colon protection and mucosal healing (Faure et al., 2006). L-arginine in intestinal ischemia animal models aids mucosal healing by reducing tissue damage (Fotiadis et al., 2016), and a similar protective effect of L-arginine was also reported in LPS (bacteria-derived lipopolysaccharide)-challenged weaned pigs (Zhu et al., 2013). Moreover, this amino acid was reported from infective stages of *N. americanus*, *N. brasiliensis*, and *T. muris* (Yeshi et al., 2020; Wangchuk et al., 2021).

Lipid utilization by helminths varies as they develop from free-living to the parasitic stage, and considerable changes occur, including lipid abundance and composition (Wang et al., 2020) (e.g., decreased synthesis of triradylglycerols but increased glycerophospholipids (Phosphatidylcholine, PC and phosphatidylethanolamine, PE in *Haemonchus contortus*)) (Wang et al., 2018), energy metabolism (Harder, 2016; Mangmee et al., 2020) and membrane fatty acids (FAs) composition (Proudfoot et al., 1990). For instance, the free-living stage requires more unsaturated FAs in the cell membrane to protect against the effects of low environmental temperatures, but the demand for FAs would be less as they develop to the parasitic stage (Hazel and Williams, 1990; Wang et al., 2018; Mangmee et al., 2020; Wang et al., 2020). Moreover, unlike the free-living infective stage, adults (parasitic stage) metabolise exogenous substrate, relying less on endogenous food stores (Barrett, 1968). In that way, adults use only a few specific FAs and fat-soluble vitamins as energy (Tielens, 1997; Dalton et al., 2003), and many lipids are eventually stored and excreted (Cheng, 1986) or become food for gametes (Barrett, 1968). We assumed that SCFAs such as propionate and acetate in the ESPs of adult *A. caninum*, *N. brasiliensis*, and *T. muris* might be stored as food reserves for their gametes. Fatty acids also play an essential role in diverse biological processes, and most importantly, FAs such as the *cis*-form of octadecanoic acid (stearic acid), monoenoic acids (oleic acid and vaccenic acid), and other branched-chain acids are known to help in breaking through the host cell membrane (Ward, 1982). Probably, the presence of stearic acid and oleic acid in the ESPs or somatic tissues of various helminth species, including *Brugia malayi*, *Dipylidium caninum*, *Dictyocaulus viviparus*, *N. brasiliensis*, *Strongyloides ratti, Toxocara canis*, and *T. muris* (Smith et al., 1996; Ferreira et al., 2014; Becker et al., 2017; Wangchuk et al., 2019; Wangchuk et al., 2019; Wangchuk et al., 2020; Yeshi et al., 2020) suggest that these worms might be producing compounds that could help them in invasion and establishing infection in their respective host(s) (Yeshi et al., 2020). Lipids are also known to have important functions in signalling transition. Endogenous steroidal hormones, such as dafachronic acid, have been demonstrated to play essential roles in regulating larval development in parasitic nematodes (e.g., in *Ancylostoma ceylanicum*, *Strongyloides stercolis*, and *Haemonchus contortus*) (Wang et al., 2009; Stoltzfus et al., 2012; Zhi et al., 2012; Albarqi et al., 2016; Ma et al., 2019). Other key nematode species, including *Ascaris suum* and *Toxocara canis* also produce dafachronic acid (Ma et al., 2019).

Zaiss *et al.* (2015) reported the immunoregulatory role of an increase in the concentration of bacterial-derived SCFAs induced by the *Heligmosomoides polygyrus bakeri* infection in mice with allergic inflammation (Zaiss et al., 2015). The same study also observed the increased SCFAs in the host-parasite setting by analysing colon contents of pigs infected with *Ascaris suum* eggs and stools from human volunteers infected with *N. americanus* infective larvae (Zaiss et al., 2015). Thus, the presence of SCFAs like propionate and acetate in *A. caninum*, *N. brasiliensis*, *T. muris*, *T. canis*, and *A. lumbricoides* might have immuno-modulatory roles in their host. Short-chain fatty acids derived from other species (not worms) also possess anti-inflammatory properties (Andoh et al., 1999; Tedelind et al., 2007; Kovarik et al., 2011), and they are known to promote gut health. Phosphatidylcholines (PCs), reported as major lipids in *S. mansoni* (Ferreira et al., 2015), are also said to have similar functions. For instance, a glycoprotein ES-62 secreted by *Acanthocheilonema viteae* can protect against rheumatoid arthritis, systemic lupus erythematosus, and airway hyper-responsiveness is mainly attributed to the covalently-bonded phosphorylcholine moieties (Pineda et al., 2014; Pineda et al., 2015). However, ES-62 did not protect against autoimmune diseases like type I diabetes, multiple sclerosis, and IBD (Doonan et al., 2018). Some schistosome-derived PCs are also responsible for pathogenesis. For instance, when toll-like receptor-2 knockout (TLR2^−/−^) mice were given *S. mansoni*-derived lysophosphatidylcholine (lyso-PC), it induced eosinophil recruitment and cytokine production in a TLR2-dependent manner, suggesting that this mechanism might contribute to the pathogenesis and lethality, particularly during the chronic phase of schistosome infection (Magalhaes et al., 2010). Nadjsombati et al. (Nadjsombati et al., 2018) showed that succinate (one of the metabolites secreted/excreted by a few helminth species) given ad libitum in the drinking water could induce a Th2 immune response in the mouse intestine through the tuft cell-ILC2 circuit, which could activate its receptor SUCNR1 to initiate succinate-induced IL-13 production, which is responsible for initiating Th2 responses.

When Giera et al. (Giera et al., 2018) analyzed the lipid composition of different life cycle stages of *S. mansoni*, eggs were found enriched with prostaglandins, while the cercaria stage contains mainly resolvins. However, Salafsky et al. (Salafsky et al., 1984) proposed that prostaglandins released by cercariae into the skin after initiating penetration upon stimulation by host skin essential fatty acids might further promote penetration *via* releasing histamine. Resolvins are well known for their anti-inflammatory properties (reviewed by Moro et al. (Moro et al., 2016)) as resolvin E1 protects liver fibrosis in mice (pathogen-free Kunming mice) infected with *S. japonicum* (Qiu et al., 2014). These findings suggest that prostaglandins and resolvins might be involved in the host immune adjustment.

## Potential application of small biomarker molecules in diagnostics

Currently, there is no 100% sensitive diagnostic tool for detecting STHs infections. The WHO-recommended gold standard microscopy-based techniques (including Kato-Katz thick smear, McMaster, and flotation, which enable to count/detect parasite eggs in a fecal sample) (Khurana et al., 2021) are not adequately sensitive in low-endemic and near-elimination areas (McCarthy et al., 2012; Werkman et al., 2018). Moreover, diagnostic assays commonly used for detecting many STHs infections are considered invasive and risky as they involve collecting samples such as body fluids (e.g., stool and blood) and tissues through biopsy, requiring both special equipment and specialist, which is often a big challenge in developing countries. There has been continued effort toward developing more sensitive, low-cost, and easy-to-operate molecular diagnostics, which can be used in both endemic and low-endemicity settings, and this topic is reviewed elsewhere (Ullah et al., 2020; Khurana et al., 2021). Recent findings about the immunomodulatory roles of helminth-derived ESPs, including proteins, microRNAs, and extracellular vesicles (Hoy et al., 2014; Kammoun-Rebai et al., 2016; Eichenberger et al., 2018; Bi et al., 2021), is a paradigm shift in diagnosis/prognosis of helminthiasis and more about proteins/protein microarrays, microRNAs, and extracellular vesicles are available in these reviews (Sotillo et al., 2020; Li et al., 2021; Mu et al., 2021). An alternative potential area of diagnostic development is small biomarker molecules. Recent advances in metabolomics techniques and technologies have enabled the identification of small biomarker molecules of infections caused by helminths. Comparative analysis of metabolites profiles in samples obtained from infected and non-infected individuals/animals can be used to identify diagnostic biomarkers. For instance, Denery et al. (2010) (Denery et al., 2010) identified 14 potential candidate metabolites biomarkers, including long-chain fatty acids hexacosenoic acid and pentacosenoic acid in the plasma and serum obtained from onchocerciasis positive human volunteers from African countries. Another example of a urine biomarker is 2-methyl pentanoyl carnitine (2-MPC), and 2-MPC could be used as *A. lumbricoides*-specific urine biomarker (**Table 2**) since it gives high accuracy for detecting both infection and infection intensity (Lagatie et al., 2020). Moreover, the 2-MPC level in urine decreased significantly when infected individuals were given a single albendazole dose (400 mg) (Lagatie et al., 2020).

**Table 2:** Reported small molecules as potential diagnostic biomarkers in samples obtained from human and animals.

Helminth species infected with/used for infection and ESP collection	Life-cycle stage used for infection/ESP collection	Samples in which SM detected	Upregulated SM	Downregulated SM	References
*(a) From infected human/patients*					
*Ascaris lumbricoides*	Samples were obtained from already infected patients	Urine	2‐Methyl pentanoyl carnitine	NA	(Lagatie et al., 2020)
*Onchocerca volvulus*		Urine	*N*-Acetyltyramine-*O*,β-glucuronide	NA	(Lagatie et al., 2016)
		Urine	Cinnamoyl glycine	NA	(Wewer et al., 2021)
		Plasma, serum	Hexacosenoic acid, Pentacosenoic acid, Hydroxy-octadecenoic acid	NA	(Denery et al., 2010)
*Schistosoma haematobium*		Urine, plasma	Indolylacryloylglycine, *N*-Glycoloylganglioside GM2, Adrenochrome *O*-quinone	NA	(Adebayo et al., 2018)
*(b) From infected experimental animal models*					
*Echinostoma caproni*	Metacercariae	Mice urine	Mannitol, *p*-Cresol glucuronide, Phenylacetylglycine, Succinate, Trimethylamine, Trimethylamine-*N*-oxide	2-Ketoisocaproate, Acetate, Creatine, Hippurate, Taurine, Glucose	(Saric et al., 2008)
		Mice plasma	Acetate, Formate	Choline, Creatine, Glucose, Isoleucine, Leucine, Valine	
		Mice fecal water	5-Aminovalerate, Isoleucine, Leucine, Uracil, Valine	Acetate, Alanine, Butyrate, Glycine, Propionate	
*Fasciola hepatica*	Metacercariae	Mice urine	Aminoadipic acid	Creatinine, Hippurate	(Saric et al., 2010)
		Mice plasma	2-Ketoglutarate, Betaine, Glucose, Trimethylamine-*N*-oxide	NA	
		Mice fecal water	3-Methyl-2-oxovalerate, Butyrate	Glucose	
*Necator americanus*	L3	Hamster urine	Aminoadipic acid, *p*-Cresol-glucuronide, Phenylacetylglycine	Hippurate, 4-Hydroxyphenylactate, Dimethylamine, 4-Hydroxy-3-methyl-phenylpropionic acid	(Wang et al., 2009)
*Onchocerca ochengi*	Sample obtained already infected cattle	Bovine nodule fluid	PE(e38:4), PE(e40:8), PE(e40:9	NA	(Wewer et al., 2017)
*Schistosoma japonicum*	Cercariae	Hamster urine	Acetate, Hippurate, *p*-Cresol glucuronide, Phenylacetylglycine, Pyruvate, Trimethylamine	Dimethylamine, Lactate, Succinate, Propionate	(Wang et al., 2006)
		Mice urine	2-Keto-3-methyl-valerate, 2-Keto-isocaproate, 2-Keto-isovalerate, 3-Ureidopropionate, *p*-Cresol glucuronide, Dimethylamine, *N*-Acetyl-glycoprotein, Phenylacetylglycine, Trimethylamine, Trimethylamine-*N*-oxide	2-Oxoglutarate, 2-Hydroxyisobutyrate, 2-(4-Hydroxyphenyl) propionic acid, Acetate, Adipate, Citrate, Fumarate, Hippurate, Succinate, Taurine	(Wu et al., 2010)
		Mice plasma	Choline, Creatine, Dihydrothymine, Glycine, Glucose, Glutamate, Glutamine, Lysine, *N*-Acetyl-glycoprotein, *Scyllo*-inositol, Valine	Acetone, D-3-Hydroxybutyrate, pyruvate	
*Schistosoma mansoni*	Cercariae	Mice urine	Trimethylamine. Phenylacetylglycine, Creatine, β-Alanine, *p-*Cresol glucuronide, Pyruvate, Tryptophan	Citrate, Acetate, Hippurate, Butyrate, Malonate, Propionate, Alanine, D-3-Hydroxybutyrate, Succinate, 2-Oxoglutarate, Taurine, 2-Oxoisocaproate, 2-Oxoisovalerate	(Wang et al., 2004)
			Tryptophan, Creatine, Benzoic acid, Uric acid, Glycolic acid	Hippurate, Citrate	(Garcia-Perez et al., 2008)
		Mice urine	Hippurate, Phenylacetylglycine, 2-Oxoadipate	NA	(Li et al., 2011)
		Mice plasma	D-3-hydroxybutyrate, Glycerophosphorylcholine	NA	
		Mice feces	5-Aminovalerate	NA	
*S. japonicum, N. americanus* (co-infection)	Cercariae, L3	Hamster urine	Butyrate, Acetone, Creatine, Phenylacetylglycine, *p*-Cresol glucuronide	4-Ethylphenol, Alanine, *N*-Acetylglycoprotein, Betaine, Succinate, 3- Hydroxyphenylpropionic acid, 4- Hydroxyphenylpropionic acid, Trimethylamine-*N*-oxide, Hippurate	(Wu et al., 2010)
*Toxocara canis*	Embryonated *T. canis* eggs	Dog serum	Calcitroic acid	NA	(Zheng et al., 2019)
*(c) From helminth-derived excretory/secretory products*					
*Ancylostoma caninum*	Adult	ESPs	Succinic acid (dominant)	NA	(Wangchuk et al., 2019)
*Nippostrongylus brasiliensis*	L3	ESPs	Octadecanoic acid (dominant)	NA	(Yeshi et al., 2020)
	Adult		Succinic acid (dominant)	NA	(Wangchuk et al., 2019)
*Schistosoma mansoni*	Cercariae	ESPs	Linoleic acid and resolvins	NA	(Giera et al., 2018)
*Trichuris muris*	Adult	ESPs	Succinic acid (dominant)	NA	(Wangchuk et al., 2019)

ESPs, Excretory/secretory products; L3, Stage 3 infective larva; NA, Not Available; reported metabolites are based on changes in the metabolite abundance (infected vs non-infected/control); ‘dominant’ means are abundant when analysed statistically (e.g., applying multivariate statistics like principal component analysis, PCA); ‘Up- or downregulation’ means increase or decrease in the levels, respectively, when compared with the negative control (e.g., samples from uninfected human or animals).

Due to challenges in biomarkers from human host-parasite interactions, focus has been diverted to study this phenomenon in animal models. There are variations in the metabolite profiles in the samples obtained from animals infected with different helminth species (**Table 2**). For example, urine samples from *S. mansoni*-infected mice showed decreased levels of SCFAs such as acetate, butyrate, and propionate (Wang et al., 2004) but urine samples from *S. japonicum*-infected and *S. japonicum* and *N. americanus* co-infected hamster had increased levels of acetate and butyrate, and reduction in propionate (Wang et al., 2006; Wu et al., 2010) (**Table 2**). Such fluctuations in the SM profile could be due to the type of helminth species used for infection or duration of infection and animal models used. Some of the commonly detected increased levels of SM in samples from infected animals are microbial-related metabolites such as *p*-cresol glucuronide, trimethylamine, and phenylacetylglycine (**Table 2**). The metabolic product of *Clostridium difficile* (opportunistic mild pathogenic anaerobe), *p-*cresol, is known to be the source of *p*-cresol glucuronide (Selmer and Andrei, 2001). Thus, elevation in such metabolites/SM could be related to the impact of infection on gut-microbial homeostasis. Another secretory product of *O. volvulus* as a potential urine biomarker for river blindness is *N*-acetyltyramine-*O*,β-glucuronide (NATOG) (Globisch et al., 2013). However, NATOG alone is inadequate to discriminate between infected and control (Lagatie et al., 2016; Hotterbeekx et al., 2020), whereby considering the level of additional urine metabolites like cinnamoyl glycine can enhance the onchocerciasis assessment (Wewer et al., 2021).

Wewer et al. (2017) (Wewer et al., 2017) reported ether-linked PC and PE in *Onchocerca volvulus*, *O. ochengi*, and *Litomosoides sigmodontis* through targeted shotgun lipidomics. Their study could not detect parasite-specific lipids in the infected bovine’s (host) serum, but the fluid taken from *O. ochengi* nodules from the host contained nematode-derived ether-linked PE species [e.g., PE(e38:4), PE(e40:8), and PE(e40:9)], which could be potentially used to develop diagnostic biomarkers for river blindness (Wewer et al., 2017). Adebayo et al. (2018) (Adebayo et al., 2018) reported few putative urine metabolites (e.g., *N*-Glycoloylganglioside, adrenochrome, 3-succinoylpyridine, 1-Nitro-5,6-dihydroxy-dihydronaphthalene) from individuals with urogenital schistosomiasis (caused by *S. haematobium*) associated with bladder pathology and are worth further analysis (including MSI level 1 identification) to be used as potential biomarkers. Oxidized pterins (e.g., neopterin) are used as urine markers (e.g., for hyperphenylalaninemia) (Longo, 2009). Pterin was detected in the ESPs of the infective L3 of *N. brasiliensis* (Yeshi et al., 2020). Considering these examples of potential small biomarker molecules and those mentioned in **Table 2**, investigating other neglected helminthiases may lead to discovering more unique biomarkers to better understand the helminth infections.

## Potential application of small molecules in immunomodulatory drugs

Several small molecule immunomodulatory drugs (IMiDs) (e.g., aminosalicylates, azathioprine, 6-mercaptopurine, and methotrexate) have been historically used for the management of inflammatory conditions, including IBD (Yeshi et al., 2020). Recent studies demonstrated that hookworm-derived SM extract could suppress pathology in TNBS-induced colitis and inhibit the secretion of key inflammatory cytokines from human peripheral blood mononuclear cells (Wangchuk et al., 2019; Wangchuk et al., 2019). Excretory/secretory products from other helminth species also showed similar protection in various experimental colitis models (Ruyssers et al., 2008; Varyani et al., 2017; Bobardt et al., 2020; Wang et al., 2020). Small molecule analogs of *Acanthocheilonema viteae* secreted immunomodulatory ESP ES-62, namely ES-62-SMAs 11a and 12b, protected ovalbumin-induced Th2 response-mediated airway inflammation and lungs eosinophil infiltration in mice suggesting potential drug leads to allergies (Rzepecka et al., 2014). Dafachronic acid detected in a few species of parasitic helminths (*A. ceylanicum*, *A. suum*, *S. stercolis*, *H. contortus*, and *T. canis*) is another example of a small molecule with therapeutic potential. When methylprednisolone acetated-treated NSG mice undergoing hyperinfection with *S. stercolis* were administered 10 μM dafachronic acid orally with drinking water, it significantly reduced the worm burden in mice (Patton et al., 2018; Wang et al., 2021). Although saturated fatty acids such as palmitic acid and stearic acid were detected in all bioactive extracts, SCFAs such as acetate, propionate, and butyrate could be responsible for the observed protection (Wangchuk et al., 2019) as Tedelind et al. (2007) have reported anti-inflammatory properties associated to these SCFAs (Tedelind et al., 2007). Dodecanoic acid produced by the miracidium of *S. mansoni* is involved in host-parasite interactions, including penetration into its intermediate host (snail) (Ferreira et al., 2014; Wangchuk et al., 2021). Linoleic acid produced by cercariae of *S. mansoni*, and prostaglandins (PGE2) enriched in their eggs helps cercariae to migrate successfully inside their human hosts (Ramaswamy et al., 2000; Giera et al., 2018). Thus, with many helminth-derived SM showing various immunomodulatory/biological properties, including anti-inflammatory (**Table 1**), it has the potential to spur novel anti-inflammatory drug and IMiDs development.

Unlike bigger molecules like proteins (biologics), SM are considered ideal candidates for drug development since they are small structures, are stable, cheaper to scale up, and lack immunogenicity (Yeshi et al., 2020). Moreover, unlike biologics that typically require parenteral delivery, SM can be developed into oral dosage forms, boosting patient satisfaction and compliance (Olivera et al., 2017). A search for SM in the innovative therapeutic design and development is still in the picture, and as of 2012, the Chemical Universe Database GDB-17 has recorded 166 billion SM (Ruddigkeit et al., 2012). Moreover, recently, a Sweden-based biopharmaceutical company, Nuevolution, has revolutionized drug discovery by creating a DNA-encoded library of 40 trillion unique SM (Makurvet, 2021). Such a library with DNA-encoded SM has enabled rapid evaluation and identification of bioactive drug-like compounds (Kleiner et al., 2011; Kunig et al., 2018). For example, a benzoxazepinone inhibitor discovered from a DNA-encoded library has become a clinical candidate that can potentially treat multiple inflammatory conditions (Harris et al., 2017). Therefore, there is an urgent need to enhance efforts to analyse helminth ESPs for bioactive molecules and establishing a helminth-specific DNA-encoded SM library would help revolutionize the development of novel therapeutics for neglected helminthiases.

## Conclusion

Recent studies have reported the protective role of small molecule crude extract obtained from helminth ES products against allergic and various autoimmune diseases, including chemically-induced colitis experimental mice models. Focusing on isolating compounds from such bioactive crude ES products of helminths would likely result in discovering novel drug leads, especially against inflammatory diseases. Limited known SM (e.g., amino acids and SCFAs) were reported from many helminths, and these molecules are associated with various biological and pharmacological activities. The available literature reports only limited known metabolites present in the crude extracts, and the identity of many metabolites remain unknown due to limitations in the identification protocols and helminth-specific compound libraries. Many of these unknown metabolites, particularly lipids that remain largely unstudied, may likely have interesting biological and pharmacological properties, which could facilitate the development of novel diagnostic and therapeutic products.

There is a real need to pursue extensive and comprehensive metabolomics studies to identify such unknown compounds. With the rapid improvement in metabolomics platforms such as high-resolution mass spectrometry (HRMS) and other associated software tools and growing databases, it is expected to accelerate the identification of many novel immunomodulatory SM produced by helminths. For example, a new pulsed mass-spectrometry ion generation technique called triboelectric nanogenerator inductive nano-electrospray ionization (TENGi nanoESI) mass spectrometry, introduced in 2020 could analyse volume-limited samples even at nanolitre scale. Such technological advancement is likely to help popularise immuno-metabolomics studies of helminths, where obtaining even modest quantities of ESPs is challenging without a good rodent model. Furthermore, future studies should prioritize those parasitic helminths not studied so far and develop a small molecule database (i.e., helminth-specific) to allow researchers to dive deep into the treasure trove of helminth metabolites.

## Author contributions

All authors contributed to the article and approved the submitted version.

## Funding

This work was funded by a James Cook University Postgraduate Research Scholarship (JCUPRS) to KY, the National Health and Medical Research Council (NHMRC) Ideas Grant (APP1183323) to PW, and NHMRC Program Grant (APP1132975) and a Senior Principal Research Fellowship (APP1117504) to AL.

## Conflict of interest

The authors declare that the research was conducted in the absence of any commercial or financial relationships that could be construed as a potential conflict of interest.

## Publisher’s note

All claims expressed in this article are solely those of the authors and do not necessarily represent those of their affiliated organizations, or those of the publisher, the editors and the reviewers. Any product that may be evaluated in this article, or claim that may be made by its manufacturer, is not guaranteed or endorsed by the publisher.
